# DNA Methylation in Ovarian Cancer Susceptibility

**DOI:** 10.3390/cancers13010108

**Published:** 2020-12-31

**Authors:** Brett M. Reid, Brooke L. Fridley

**Affiliations:** 1 Department of Cancer Epidemiology, H. Lee Moffitt Cancer Center, Tampa, FL 33612, USA; brett.reid@moffitt.org; 2 Department of Biostatistics and Bioinformatics, H. Lee Moffitt Cancer Center, Tampa, FL 33612, USA

**Keywords:** DNA methylation, epigenetics, epimutations, ovarian cancer, genetic susceptibility, meQTL, epigenome-wide association studies, genome-wide association studies

## Abstract

**Simple Summary:**

It is well established that ovarian cancer “runs in families”, where ovarian and other cancers (commonly breast cancer) occur at early ages at onset and in multiple generations. After decades of genetic studies, rare high-risk genetic mutations in cancer susceptibility genes and over 40 common genetic variants with much smaller risks have been identified. However, based on familial studies, we know that additional heritable genetic risk factors exist. It is possible that epigenetic variation—differences in how DNA is read, and which genes are actively expressed (or not) —also contributes to ovarian cancer susceptibility. This review summarizes the current collection of epidemiological studies that have investigated the role of DNA methylation—one type of epigenetic mechanism—in the risk of ovarian cancer.

**Abstract:**

Epigenetic alterations are somatically acquired over the lifetime and during neoplastic transformation but may also be inherited as widespread ‘constitutional’ alterations in normal tissues that can cause cancer predisposition. Epithelial ovarian cancer (EOC) has an established genetic susceptibility and mounting epidemiological evidence demonstrates that DNA methylation (DNAm) intermediates as well as independently contributes to risk. Targeted studies of known EOC susceptibility genes (CSGs) indicate rare, constitutional *BRCA1* promoter methylation increases familial and sporadic EOC risk. Blood-based epigenome-wide association studies (EWAS) for EOC have detected a total of 2846 differentially methylated probes (DMPs) with 71 genes replicated across studies despite significant heterogeneity. While EWAS detect both symptomatic and etiologic DMPs, adjustments and analytic techniques may enrich risk associations, as evidenced by the detection of dysregulated methylation of *BNC2*—a known CSG identified by genome-wide associations studies (GWAS). Integrative genetic–epigenetic approaches have mapped methylation quantitative trait loci (meQTL) to EOC risk, revealing DNAm variations that are associated with nine GWAS loci and, further, one novel risk locus. Increasing efforts to mapping epigenome variation across populations and cell types will be key to decoding both the genomic and epigenomic causal pathways to EOC.

## 1. Introduction

Epithelial ovarian cancer (EOC) is a relatively rare cancer with a large heritable component that presents an opportunity for genetic risk prediction [1] to inform early intervention as well as therapeutic strategies. First-degree relatives of women with EOC have an approximately three-fold higher risk of developing the disease and this can increase up to 10-fold when multiple relatives are affected [2]. Genetic epidemiology studies have identified a range of susceptibility alleles across the allelic spectrum from rare, high penetrance variants in *BRCA1* and *BRCA2* to uncommon, moderate penetrance (e.g., *BRIP1*, *RAD51C/D*, *MSH6*) and over 40 common, low penetrance variants from genome-wide association studies [3]. Collectively, known genetic variants account for 45% of the estimated EOC heritability [3] and the remaining portion may be explained by other sources of heritability. Epigenetic variation contributes to phenotypic variance, evolution, and complex trait heritability [4] and could play an important role in EOC susceptibility.

The concept of epigenetics was first introduced in 1942 [5] to explain changes in cellular phenotypes during organismal development. Since then, epigenetics has grown to encompass a broad catalogue of biological mechanisms and molecules that control DNA-template processes such as transcription, replication, and repair, to establish and maintain a wide range of cellular phenotypes and physiological states [6]. Mechanistically this is achieved by altering the organization and function of chromatin through the plastic layering of post-translational modifications to DNA and histones, as well as non-coding RNA interference [7,8]. The most studied and well-understood epigenetic mechanism is DNA methylation (DNAm), whose critical role in carcinogenesis was first recognized almost 40 years ago [9]. As the most stable and readily measured—particularly with relatively low-cost microarray technology—DNAm is the only mechanism that has been evaluated in human population studies thus far [10,11].

Over the years an extensive collection of epigenetic literature has been amassed and several lines of evidence now demonstrate that inheritance of epigenetic information influences metabolic and behavioral states and disease [12]. Recently, the transgenerational epigenetic inheritance of cancer susceptibility was functionally authenticated in a mouse model where the deletion of chromatin regulator *KDM6A* in the paternal germ line increased tumor incidence in genetically wild type progeny [13]. Furthermore, DNAm changes in mutant sperm were found to be retained in the somatic tissue of wild-type progeny. Moving forward, observational quantitative genetic and epigenetic studies will be key to determining if this is a representative model of epigenetic inheritance for cancer in human populations.

Here, we provide a review of the epidemiological studies that have evaluated DNAm in association with EOC susceptibility. As a preface, we briefly summarize the molecular and population features of DNA methylation and their known role in cancer development and heritability.

## 2. DNA Methylation in Epigenetic Control and Heritability

In mammals, DNA modifications predominantly occur through cytosine methylation (CH_3_) at guanine-cytosine dinucleotides (CpG) which cluster in CpG-dense regions called CpG islands (CGI) [14]. A critical role of CpG DNAm is the regulation of gene expression through transcriptional silencing, an essential mechanism for developmental processes, including parent-of-origin imprinting, X-chromosome inactivation, and transposon silencing, and cellular differentiation [14]. For each cell, DNAm is inherited through successive cell cycles resulting in cell type patterns that extend through a specific lineage [15]. Cellular identity is responsive to numerous spatiotemporal factors including environmental cues (e.g., stimuli, signaling), cell cycle phase, and physical environment (e.g., oxygen availability, anchoring) [16]. Thus, while DNAm are stable epigenetic marks that establish a cellular memory and control of phenotype, they are not static. Recent studies mapping DNAm across cell types and populations has revealed cell-type specific patterns of variable methylated regions (VMRs) with high inter-individual variation [17].

The loss of epigenetic control over cellular identity and physiological homeostasis is an enabling hallmark of cancer. Oncogenic transformation is achieved through the cooperation of genetic and epigenetic reprogramming [18] to alter gene expression resulting in widespread DNAm alterations, often called epimutations [19]. Methylation across a variety of tumors show patterns of global hypomethylation as well as regions of focal hypermethylation at normally unmethylated CGI, often in the promoters of tumor suppressor and DNA mismatch repair genes [20]. EOC presents similar patterns but with histotype specificity [21,22]. High-grade serous ovarian cancers (HGSOCs) exhibit few hypermethylation events, although *BRCA1* hypermethylation has been observed in 11.5% [23]. Conversely, endometrioid ovarian cancer (ENOC) is characterized by frequent promoter hypermethylation (CpG island methylation phenotype, CIMP) that is also common in uterine endometrioid tumors and parsimonious with the hypothesized endometriosis origin of ENOC [24]. Clear cell ovarian cancer (CCOC) also distinguishes itself by displaying the highest frequency (>70%) of hypermethylation [25]. It is important to note that differences in tumor DNAm may be associated with response to chemotherapy and prognosis. Several studies have investigated DNAm in association with treatment response and prognosis, and have been reviewed elsewhere [22].

Under normal conditions, DNAm acts as a critical barrier to cellular reprogramming and tumor initiation [26]. In normal tissues, aberrant DNAm can occur as a result of both somatic epimutations that arise and accumulate during the lifespan [27,28] and wide-spread ‘constitutional’ epimutations that originate in the parental germline or during early embryogenesis [29]. While most methylation is removed from the parental germline during embryogenesis, experimental studies have demonstrated a significant level can be retained across generations by resisting erasure [13,30] and through regulation of de novo methylation by non-coding RNA (ncRNA) populations packaged in sperm and oocytes [31].

**Figure 1:**
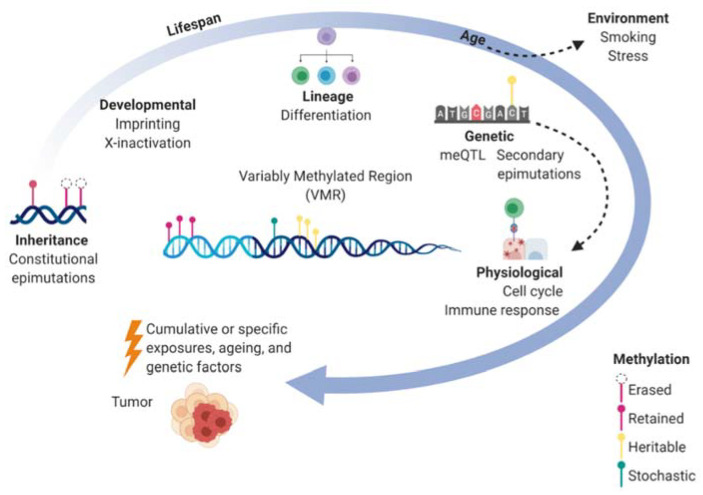
Dynamics of the methylome over the lifespan and the contribution to cancer. Within a variably methylated region (VMR) for a particular cell, the methylome can be shaped by molecular factors (inside circle) such as inherited methylation marks, developmental processes, cellular differentiation, genetic sequence, and physiological states which are shown to be dependent on genetics as well. Environmental factors also effect methylation where epimutations not only accumulate over time but have larger effects on methylation with ageing. The cumulative effects of genetic, epigenetic, and environmental exposures contribute to the development of cancer.

During the lifespan, DNAm is continuously influenced by environmental exposures and genetic sequence as well as interacting factors such as age and gender [32]. Genetic variants are estimated to account for ~20% of the inter-individual variance in methylation though each CpG site varies in heritability and some (18%) have shown as high as >99% genetic heritability [32]. Constitutional epimutations that occur in association with genetic variants are considered secondary epimutations. Adding further complexity, CpG heritability has been shown to vary over time where environmental factors contribute more to methylation variance with increasing age. Genetic effects on DNAm are also associated with transcriptional response such as activation of immune genes [33] meaning meQTL vary depending on physiological states. Thus, the methylome of an individual at any particular VMR is a function of a multitude of factors that are dynamic over the lifespan and can cumulatively or specifically contribute to cancer (Figure 1). The heritable components of the methylome (both epigenetic and genetic) could contribute to the familial heritability of EOC.

In the following sections, we present a review of the epidemiological studies that have explored DNAm and EOC susceptibility. These have been categorized by study design and include: (1) familial and population-based studies targeting known cancer susceptibility genes (CSGs), (2) epigenome-wide association studies (EWAS), (3) integrative genomic-epigenomic approaches to study DNAm underlying genetic associations with risk, and (4) environmental epigenetic studies evaluating DNA methylation as a mediator of environmental risk.

## 3. DNA Methylation in Ovarian Cancer Susceptibility Genes

Both primary and secondary constitutional epimutations that contribute to cancer susceptibility have been definitively described in CSGs for Lynch syndrome (*MLH1*, *MSH2*) and Wilms Tumor (*H19*) [29], but other cancers are also emerging such as colorectal cancer (*MGMT*) and breast cancer (*BRCA1*) [11]. Four studies have evaluated DNAm to identify constitutional epimutations in known CSGs that contribute to EOC susceptibility: one case-only study [34], two case-control studies [35,36], and one familial segregation study [37] (Table 1). These studies limited cases to women without *BRCA1* or *BRCA2* sequence mutations to enrich the study population for non-genetic mechanisms. Three studies additionally limited cases to familial and/or early-onset EOC [34,36,37] while the other did not have family history criterion [35]. From peripheral blood leukocytes (PBL), DNAm in gene promoter regions was quantified via bisulfite sequencing technologies. Bisulfite pyrosequencing has been the standard practice for hypermethylation detection in clinical epigenetic studies and is generally followed by clonal or plasmid bisulfite sequencing for validation and confirmation of allele-specific methylation [38].

All four studies evaluated *BRCA1* and together suggest *BRCA1* promoter hypermethylation could be associated with EOC susceptibility as both constitutional primary and secondary epimutations. In a familial segregation study of families with breast and ovarian cancer, *BRCA1* promoter hypermethylation was present in two out of 49 families, including one proband with triple negative breast cancer and one with HGSOC [37]. DNA sequencing identified a 5′ UTR *BRCA1* variant that segregated with *BRCA1* hypermethylation following a dominant inheritance pattern suggesting a secondary constitutive epimutation. In another study, promoter hypermethylation of *BRCA1* was observed in 8% (three in 39) of cases with EOC and 1% (six in 613) of cases with breast cancer but in none of 10 age-matched controls [34]. It was further demonstrated that *BRCA1* hypermethylation was confined to a single parental allele, present in all fractions of myeloid cell types (mesoderm) as well as urine (endoderm), and did not correlate with local DNA sequence variants. Together, these findings aligned with the definition of a primary constitutive epimutation although correlated sequence variation may still underlie the hypermethylation observed. This study also detected *RAD51C* promoter hypermethylation in one case of EOC.

Methylation of *BRCA1* has been further implicated in a large-scale, two-stage study totaling 1640 EOC cases and 3682 controls where promoter methylation was detected in 6.4% of EOC cases compared to 4.2% of controls, and was associated with increased risk of EOC (OR = 1.83, 95% CI = 1.27–2.63) [35]. The study also included histotype-specific analysis which has not been reported elsewhere, finding that increased prevalence of in *BRCA1* promoter methylation was specific to HGSOC and not significantly higher in LGSOC or non-serous histotypes. Sensitivity analyses showed that methylation frequency did not differ by cancer stage, surgery (before vs. after), chemotherapy, or tissue storage time but did decrease with age which was adjusted for in their comparisons. Additionally, tumor *BRCA1* promoter methylation was five-times more frequent in cases with PBL methylation (18 in 29, 62%) than in cases without (seven in 58, 12%). A substantial proportion (54%, 13 in 24) of cases with PBL *BRCA1* methylation also displayed methylation amongst other organ tissues whereas none of the patients without PBL methylation showed this feature, suggesting inheritance of the epimutation rather than somatic origin.

In contradiction to these findings, a study of 108 patients with breast and/or ovarian cancer and 60 controls found no subjects with promoter hypermethylation in *BRCA1* [36]. The discrepancy in detection may be due to dissimilarities in study design since they did not age-match controls that were significantly younger (median age 61 vs. 41 in cases and controls, respectively) or limit to chemo naïve blood samples. They also employed different methylation quantification technology and analytical methods which can both contribute to differential detection. 

In summary, the current literature demonstrates the prevalence of constitutional epimutations in *BRCA1* that may well contribute to EOC susceptibility. However additional large-scale, confirmatory studies are needed. Further studies with histotype-specific analysis are particularly warranted given the observed specificity for HGSOC thus far. Finally, it is important to note that it is unknown whether the promoter hypermethylation originates in the early zygote or germline. A recent study evaluated *BRCA1* promoter methylation in mother–daughter pairs [39]. However, these data allow no conclusions to be made on maternal transfer [40].

## 4. Epigenome-Wide Association Studies of DNA Methylation

Genome-wide DNAm has been examined in four case-control studies termed epigenome-wide association studies (EWAS) (Table 2). Blood samples were retrospectively collected and profiled using methylation microarrays with single CpG site probes [41] to identify differentially methylated probes (DMPs). DMPs may be symptomatic—caused by immune or other reactive response to the disease state—or etiologic differences that contributed to disease development. It is not possible to distinguish between the two within the retrospective design, making it difficult to discern the implications for EOC susceptibility. Nevertheless, EWAS have generated a significant compilation of EOC-associated PBL epimutations as well as insight into important dynamics of blood-based DNAm and useful analytical strategies.

The first EWAS in EOC compared 25,642 CpG probes between 113 pre-treatment cases and 148 unaffected controls and detected 2714 DMPs [42]. With just the top 100 DMPs, EOC cases could be accurately predicted (AUC = 0.80, 95% CI = 0.84–0.87). It also performed well for post-treatment cases with active disease (AUC = 0.76, 95% CI = 0.72–0.81) and poorly for those without active disease (AUC = 0.52, 95% CI = 0.48–0.55). Further analysis showed that the DMPs largely overlapped with age-related DMPs and strongly correlated with cell type distributions. Systematic differences in the leukocyte sub-populations were later confirmed as the major contributor of the findings when the ~2700 DMPs were shown to be highly correlated with leukocyte-tagging DMPs (spearman correlation = 0.75) and only 17 DMPs were non-leukocyte DMPs [43,44]. Based on this evidence, subsequent studies of PBL DNAm have used a series of analytical methods to mitigate confounding by cell-type.

Two additional EWAS studies were performed in parallel using the same hospital-based population but employing different analytical methods to mitigate confounding by cell-type. One study analyzed the total available sample size of 336 EOC cases and 398 controls but limited analysis to ~14,000 probes that were not previously associated with leukocyte cell-types (i.e., probe filtering) [45]. The other analyzed a subset of the same population (242 cases/181 controls) that had complete blood count (CBC) measures available for direct adjustment of cell-type distribution within logistic regression models. Though the sample size was reduced, this approach allowed more (~22k) CpG probes to be assessed [46].

While both methods aimed to accomplish the same goal—identification of DMPs that were independent of cell-type differences—the results were strikingly disparate with no overlap between findings. With the probe filtering method [45], 30 DMPs were detected and pathway analysis implicated enrichment for the telomerase signaling pathway (*HDAC3, IL2RG, PIK3C2B, PIK3R1*, and *POT1*) and paxillin signaling pathway (*ARFIP2, ITGB6, PIK3C2B, PIK3R1,* and *SRC*). Other findings relevant to cancer biology included hypomethylation in the gene body of *HHIP* (hedgehog-interacting protein) which has been associated with tumor growth and angiogenesis [47], and promoter hypomethylation for antiapoptotic *CUL7* that inhibits p53 [48] and Caspase-8 [49]. With the CBC adjustment method [46], 62 DMPs were identified with the most significant local to cancer-associated genes *SOCS2* and *SEPT9*, the latter of which has been previously implicated in ovarian tumorigenesis [50]. Notably, 61 (98%) of the CBC-adjusted DMPs overlapped with previously reported unadjusted DMPs from Teschendorff et al. (2009), suggesting that the replicated probes are either robust to cell-type distribution or that the CBC adjustment method was not able to remove all confounding by cell-type. In the filtering method, only six (20%) DMPs overlapped with prior unadjusted results. However, the complete lack of overlap between filtered and CBC-adjusted probes in the sample clinical population suggests these may be leukocyte-associated DMPs.

The study by Winham et al. [46] also performed novel regional-based testing for a subset (n = 163) of samples profiled using a higher density array. CpG probes were aggregated into 25,607 CpG islands to increase statistical power but this method also has biological relevance since individual CpG methylation is less stable and usually the entire promoter is either methylated or not [51]. Interestingly, highly ranked single DMP sites were not significant at the regional-level based testing and vice versa. Rather, additional regions were identified that corresponded with known CSGs including the CpG island in the promoter of *BNC2*, a CSG identified through GWAS [52]. Another region identified was in the promoter of *XRCC2*, a homologous recombination gene that has been indicated in EOC susceptibility and other cancers [53].

Another EWAS study was recently conducted among Chinese women and differed from prior studies by using a two-stage design [54]. The initial discovery stage compared methylation across ~485,000 CpG probes in 24 EOC cases and 24 controls and validated 96 DMPs in an independent set of 205 EOC cases and 205 controls. In total, 40 validated DMPs were identified which where enrichment for immune process genes (e.g., *LYST*, *CADM1*, *NFATC1*). The significant probes did not show correlation with leukocytes. However, they did correlate with platelet count and coagulation factors. Only 16 DMPs were not associated with platelets or coagulation factors which included hypermethylation of *CADM1* and *CADM2* and hypomethylation of *LYST*. *CADM1* is known to be a tumor suppressor gene for solid tumors [55] including EOC [56] and is frequently inactivated by promoter hypermethylation [57]. Observed expression of *CADM1* was lower in blood cells of EOC cases possibly caused by promoter methylation. *LYST* is a lysosomal trafficking regulator that promotes proliferation and inhibits apoptosis in multiple myeloma [58], and also harbors driver mutations for rare bone tumors [59]. Notably, this is the only EWAS study that has analyzed DNAm by EOC histotypes. In the histotype-specific analysis, most DMPs were associated with serous and endometrioid EOC while 10 were associated with mucinous EOC, in concordance with a distinct etiology for mucinous EOC [3]. DMPs that were only significant in histotype-specific analysis included three serous (*LYST*, *SUN1*, *C9orf92*), two endometrioid (*SAMHD1*, *GLRX2*), and one mucinous and endometrioid CpG probe (*CD177*).

In total, EWAS studies have analyzed a total of 801 EOC cases and 715 controls and identified 2846 DMPs (Table S1). We observed little replication across studies largely owing to confounding by blood-level factors (cell type distribution, coagulation factors) but also study heterogeneity. While most EOC cases were comprised of serous histology they also included a wide range (28–51%) of non-serous ovarian cancers causing phenotypic heterogeneity across studies. Although histotype-specific DMPs were only reported in one study, the findings indicate PBL DNAm may display unique epigenetic alterations that occur during tumorigenesis [21]. EWAS studies also included adjustments for different confounders such as demographic and known EOC risk factors (age, parity, age of first birth, alcohol use, smoking, enrollment year/state) [45,46] and experimental factors (bisulfite conversion efficiency, array batch) [42]. Despite these differences, 82 DMPs were replicated in at least one other study comprising 71 replicated genes (Table S2). It is conceivable that some of the cancer-associated probes could also include predisposition or risk-associated DMPs. As we noted, several known tumor suppressor genes and oncogenes have been identified across EWAS and represent candidate susceptibility genes.

## 5. Genetic Susceptibility Mediated by DNA Methylation

The functional connection between genetic and epigenetic variation is an integral component of the genetic predisposition to cancer [60]. Numerous studies have mapped genetic variation to CpG methylation levels (methylation quantitative trait loci, meQTL) and shown these associations underlie DNAm variation and the inheritance of complex traits and diseases [33,61]. Three studies have explored methylation-mediated relationships between genetic variation and EOC risk using different integrative approaches.

Shen et al. [62] performed a candidate gene study and comprehensive analysis of tumor DNAm, associated meQTL, and gene expression for *HNF1B*, a suspected susceptibility gene from tumor methylation patterns. *HNF1B* was hypermethylated in approximately 50% of HGSOC tumors (n = 608) but not in any of the CCOC tumors profiled (n = 4). Among SNPs within 150kb of *HNF1B*, nine SNPs were associated with increased risk for HGSOC (rs7405776 OR = 1.13, 95% CI = 1.09−1.17, P = 3.1 × 10^−10^) but conversely were associated with reduced risk for CCOC (rs11651755 OR = 0.77, 95% CI = 0.70−0.84, P = 1.6 × 10^−8^) (Table 3).

Four of the risk SNPs in the 5′ UTR of *HNF1B* were significantly correlated with promoter CpG methylation in tumors, suggesting the aberrant DNAm observed in tumors could be etiologic. Immunohistochemical (IHC) analysis further showed that the HFN1B protein was expressed in most CCOC tumors where the *HNF1B* promoter was not methylated whereas the majority of HGSOC did not express the HNF1B protein and had frequent *HNF1B* promoter methylation. Together, these findings demonstrated that risk-associated variation in *HNF1B* alters promoter methylation for HGSOC and CCOC in opposing directions, suggesting it may have a tumor suppressor role in HGSOC and a reverse, oncogenic role in CCOC.

Two genome-wide studies have evaluated PBL DNAm and genetic variation in EOC but used different analytical frameworks [63,64]. One was an EWAS-based approach that identified DMPs and then evaluated whether they correlated with risk SNPs [63]. For the EWAS, PBL CpG DNAm was profiled and compared between 214 cases and 214 controls and identified 1993 DMPs that were subsequently filtered to 185 DMPs with meQTL. Twenty-eight of the meQTL were associated with risk and mediation analysis using the causal inference test [65] revealed that 13 DMPs modulated associations of 17 SNPs. Interestingly, prior EWAS studies [42,46] also detected DMPs at *AIM2* and *STAB1* which could both conceivably affect immune system response and cancer development. *AIM2* is a member of innate immune sensors that initiate inflammasomes and trigger secretion of proinflammatory cytokines [66]. Although it has displayed both tumor suppression and promotion roles across cancer types, its upregulation in ovarian cancers suggests it could promote EOC progression [67,68]. *STAB1* is a transmembrane receptor expressed on macrophages and lymphatic endothelial cells that acts as an inhibitor of antitumor immunity [69].

It is also notable that all meQTL that linked to the 13 DMPs were *trans* associations where the SNP was located on a different chromosome than the DMP. This was also true for the entirety of the 427 DMP-meQTL pairs identified. It is surprising that *trans*-meQTL, rather than *cis*-meQTL, were strictly observed for DMPs since they are estimated to account for a small portion of heritable PBL CpG (7% vs. 73%, respectively) [70]. Furthermore, VMRs within the genome have shown to be correlated in *cis* and *trans* forming co-methylated networks with low genetic heritability and high cell type specificity [17]. Thus, the predominance of *trans*-meQTL is intriguing and it is provocative to postulate whether it’s a reflection of differential blood cell distributions or some other underlying biology.

The second genome-wide study used a GWAS-based approach that imputed and compared genetically inherited PBL DNAm levels between 22,406 EOC cases and 40,941 controls [64]. In this innovative application, meQTL were used as genetic instruments to estimate CpG methylation levels, effectively removing both symptomatic and confounding differences in methylation that are present in typical EWAS. High density genetic and PBL DNAm data from the Framingham Offspring Study [71] were used to build genetic prediction models and risk associations for 62,938 CpG probes were estimated from 751,031 SNPs using GWAS summary statistics. A resulting 89 differentially methylated CpG were significantly associated with EOC risk and included eight known genomic risk regions where seven were correlated with local gene expression (Table 3). Additionally, one novel risk region was identified where increased risk of EOC was associated with hypermethylation of two CpG sites that were correlated with reduced expression of *ADAP1* (7p22.3) a GTPase-activating protein that functions as a scaffold in several signal transduction pathways. Histotype-specific analysis showed that all DMPs were associated with HGSOC and three loci (3q25, 17q21.31, and 19p13.11) had HGSOC-specific DMPs. The 2q31.1 locus was the only region that was also significant for mucinous and endometrioid histotypes.

In comparison to prior EWAS study findings, the integrative GWAS-based approach replicated (*p* < 0.10) EWAS associations with increased risk of EOC for hypomethylation of cg19399532 at *C1orf220* [42,46] and hypomethylation of cg21870884 at *GPR25* [46]. *C1orf220* is a long non-coding RNA that has exhibited upregulation in lung squamous cell carcinoma [72] and was one of the 71 genes that was replicated across EWAS studies and did not correlate with age. *GPR25* is a G-protein coupled receptor that activates signaling cascades as a response to extracellular stress and has been linked to heritable arterial stiffness [73].

Collectively, variation in DNAm has been mapped to nine GWAS risk loci using integrative approaches for tumor and PBL DNAm (Table 3). Future studies mapping meQTL in additional tissue and cell types will be an important undertaking to further elucidate the known genetic susceptibility as well as identify additional risk loci. Currently, meQTL mapping and imputation have been performed with *in cis* SNPs but it will be intriguing to investigate the relationship of *trans*-meQTL with EOC risk using this approach.

## 6. Environmental Risk Mediated by DNA Methylation

In the prior section, the association between DNAm and EOC susceptibility was evaluated within a framework focused on the mediation of genetic effects on phenotypic variance, i.e., genetic (GWAS SNP) → epigenetic (meQTL association) → phenotype (EOC). However, DNAm may also be a mediator of environmental exposures whereby DNAm alterations can occur without DNA sequence alterations and these changes can influence gene expression and hence EOC susceptibility, i.e., environmental exposure (risk factor) → epigenetic (DMP) → phenotype (EOC). Thus far, only one study has evaluated DNAm as a mediator of an environmental risk factor for EOC.

In the study by Wu et al. [74], DNAm was evaluated as a causal mediator of the association between EOC risk and alcohol use which is an exposure known to alter DNAm [75]. Among 196 EOC cases and 202 controls previously analyzed in prior EWAS [45,46,63], DNAm was evaluated in combination with alcohol consumption. Approximately 63% of cases and 85% of controls reported alcohol use at study enrollment which was associated with a significant inverse association with EOC risk (OR = 0.34, *p* = 0.001). Alcohol use was associated with reduced CpG methylation in both cases and controls for two DMPs on chromosome 11 that were found to be significant mediators of the association between alcohol consumption and reduced EOC risk: cg09358725 at *LMO2* and cg11016563 at *TRPC6*. While this study suggests alcohol use may associate with EOC status by regulating CpG-specific DNAm patterns, effects on DNAm may be temporary and the identified DMPs may not be stable. Though results should be interpreted cautiously, they are encouraging for additional studies to validate these findings.

Studies are needed to assess DNAm as a mediator of other known EOC risk factors. DNAm alterations have been associated with several previously reported EOC risk factors, including sex hormone exposure [76], obesity [77], endometriosis [78], irregular menstruation in women with polycystic ovarian syndrome [79], as well as perceived stress, cortisol output, and inflammation [80]. Integrating epigenetics into epidemiological investigations of these exposures may help to elucidate etiological mechanisms of this disease. Furthermore, future studies should also consider whether DNAm may be a modifier of, or act in combination with (i.e., interaction), both genetic and environmental risk factors for EOC [81]. 

## 7. Conclusions

DNAm has been at the forefront of epigenetic research and has provided a paradigm for the epigenetic inheritance of cancer susceptibility. The complexities of not only the inheritance but the dynamics of DNAm across cell types and the lifespan have presented challenges to the investigation and interpretation of epimutations. Nevertheless, a growing collection of epidemiological studies with various designs have begun to elucidate the role of DNAm in EOC susceptibility. While rare hypermethylation occurrences have been observed, phenotypic variance associated with differences in DNAm secondary to genetic variation appears to be a significant component of the heritability of EOC. Across EWAS and integrative study designs, 25% (n = 10) of GWAS risk loci have been correlated with DNAm changes. Notably, a novel genome-wide significant DNAm risk locus has also been detected. As detailed maps of DNAm variation across populations and tissues, and in linkage to methylation (meQTL), expression (eQTL), and transcriptional response (reQTL) continue to be generated, further revelations of not only individual genetic–epigenetic loci but potentially co-methylated networks are expected to follow.

While studies with genetic–epigenetic approaches are increasing, studies that integrate DNAm with environmental exposure data are comparably lacking. More research is needed to elucidate the relationship between epigenetics and environmental exposures and how they contribute to the heritability of EOC. Truly comprehensive evaluations of DNAm will require integration with both genetic and environmental factors. This multi-dimensional data will carry a higher computational burden necessitating larger sample sizes. To-date, EOC EWAS studies have been conducted in a significantly smaller number of samples (<2000 subjects overall) compared to GWAS (>100,000 subjects). Imputation-based methods for DNAm, such as those employed by Yang et al. [64], could be one approach to boost power but will require more complex imputation algorithms that account for environmental exposures and may not be available in reference datasets. Ideally, large-scale DNAm profiling initiatives should be undertaken, preferably with the higher coverage technologies that are now available.

Currently, microarrays offer coverage of over 850,000 CpG sites and targeted bisulfite sequencing is another relatively low-cost option with even higher coverage, capturing up to several million CpGs. Whole-genome bisulfite sequencing (WGBS) has typically been cost-prohibitive for association studies, however, it continues to improve in terms of efficiency and accuracy with lower input amounts and read-depths required, making it an increasingly feasible option [82]. The application of WGBS will be imperative for a more comprehensive assessment of DNAm since the coverage of cell-type-specific VMRs are not well represented with a fixed content design [83] and it captures additional DNAm features such as allele-specific methylation [84]. Overall, as technology continues to advance and costs decrease, future epigenetic studies will be able to evaluate larger numbers of samples with improved feature capture, enabling further discovery that will enhance our understanding of DNAm and its contribution to cancer susceptibility.

## Figures and Tables

**Table 1:** DNA methylation in known ovarian cancer susceptibility genes.

First Author, Year, Country	Study Design	Sample Size ^a^	Case Criterion	Platform	Methylation Type, Thresholds	Prevalence		Comments
						Gene(s)	N (%)	
Hansmann, 2012, Germany	Case-only	641 Cases (613 BC, 39 EOC)	Familial or early- onset^a^ BC or EOC Chemotherapy naïve *BRCA1*/2 negative	Bisulfite pyrosequencing (4-7 CpG) Bisulfite plasmid sequencing (27 CpG)	Hypermethylation as 75th percentile + 3xIQR, >6% for all genes	*ATM* *BRCA1* *BRCA2* *PTEN* *RAD51C* *TP53*	0 All: 9 (1.4%) EOC: 3 (8%) BC: 6 (1%) 0 0 All: 3 (0.5%) EOC: 1 (2.5%) BC: 2 (0.3%) 0	10 age-matched controls were tested for *BRCA1* and none were hypermethylated. Formal statistical comparison was not included. Urine, saliva also profiled
Lonning, 2018, Norway	Two-stage case-control	Phase I: 934 Cases, 1698 Controls (332 HGSOC, 298 LGSOC, 295 Nonserous) Phase II: 607 Cases, 1984 Controls (286 HGSOC, 151 LGSOC, 170 Nonserous)	Invasive EOC Chemotherapy naïve *BRCA1/2* negative	qPCR Bisulfite pyrosequencing (NR)	Manually scored positive/negative methylation status >Median	*BRCA1*	Controls: 70 (4.2%) All EOC: 59 (6.4%) OR=1.8 (1.27-2.63) HGSOC: 32 (9.6%) OR=2.9 (1.85-4.56) LGSOC: 12 (4.0%) OR=0.98 (0.54-1.79) Nonserous: 15 (5.1%) OR=1.23 (0.71-2.13)	932 post- and 784 pre-surgery 333 not tested for BRCA1/2 in phase II Age-matched controls Age-adjusted logistic regression Tumors also profiled
Evans, 2018,	Segregation	49 probands	Familial BC or EOC Manchester score>34 *BRCA1/2* negative	Bisulfite pyrosequencing (10 CpG) Bisulfite plasmid sequencing	Mean promoter methylation (%)	*BRCA1*	Proband: 2 (4%) ~50% methylated Dominant inheritance; segregates with c.−107A>T in the *BRCA1* 5′ UTR	*BRCA1* promoter hypermethylation was 71.4% informative Buccal mucosa, tumor, hair samples also profiled
Tabano, 2020, Italy	Case-control	108 Cases, 60 Controls	Invasive or DCIS BC or high-grade non-mucinous EOC *BRCA1/2* negative	MassARRAY^®^ EpiTYPER (9 CpG *BRCA1*, 11 CpG *RAD51C*)	Hypermethylation as >UCL 95% CI ^b^, >13.6% *BRCA1* >12.1% *RAD51C*	*BRCA1* *RAD51C*	0 Case mean: 4.4% BC: 4.3% EOC: 3.9% Control mean: 4.3% 0 Case mean: 3.7% BC: 3.7% EOC: 3.5% Control mean: 4.3%	

NR = not reported; BC = breast cancer; EOC = epithelial ovarian cancer; BC = breast cancer; HGSOC = high-grade serous ovarian cancer; LGSOC = low-grade serous ovarian cancer; PBL = peripheral blood leukocyte; qPCR = quantitative polymerase chain reaction; OR = odds ratio. ^a^ Histotype-specific sample size included when reported in study. ^b^ Age at diagnosis < 51 years. ^c^ 1000 bootstrap samples in controls was performed to derive the one-sided 95% percentile bootstrap confidence interval of the controls’ mean.

**Table 2:** Epigenome-wide association studies of DNA methylation in ovarian cancer.

First Author, Year, Region	Study Design, Population, Ancestry	Cases/ Controls	Histology, N (%) ^a^	Platform (No. Cpg Tested)	Results		Probe/Gene Replicated
					DMP	AUC (95% CI) ^b^	
Teschendorff, 2009, UK	Retrospective, Population-based, European	235/148	Serous, 152 (57%) Endometrioid, 37, (14%) Mucinous, 30 (11%) Clear Cell, 28 (11%) Other, 19 (7%)	HumanMethylation27 (25,642)	2714	0.80 (0.74–0.87) ^c^ 0.76 (0.72–0.81) ^d^	73/71
Fridley, 2014, USA	Retrospective, Hospital-based, European	336/338	Serous, 243 (72%) Endometrioid, 63 (19%) Mucinous, 8 (2%) Clear Cell, 16 (5%) Other, 6 (2%)	HumanMethylation27 HumanMethylation450 (13,816)	30	NE	6/6
Winham, 2014, USA	Retrospective, Hospital-based, European	242/181 ^e^	Serous, 168 (70%) Endometrioid, 45 (19%) Mucinous, 7 (3%) Clear Cell, 12 (5%) Other, 10 (4%)	HumanMethylation27 HumanMethylation450 (22,278)	62	NE	61/59
Li, 2017, China	Retrospective, Hospital-based, Han Chinese	230/229	Serous, 109 (49%) Endometrioid, 59 (26%) Mucinous, 24 (11%) Other, 32 (14%)	HumanMethylation450 (450k)	Overall:		6/5
					40	0.77 (0.73–0.82)	
					Serous:		
					32	0.77 (0.71–0.83)	
					Endometrioid:		
					34	0.80 (0.74–0.86)	
					Mucinous:		
					11	0.73 (0.61–0.84)	
TOTAL		801/715	Serous, 504 (52%) Endometrioid, 267 (28%) Mucinous, 69 (7%) Clear Cell, 56 (6%) Other, 67 (7%)		2846		82/71

DMP = Differentially methylated probe; NE = not estimated; AUC = area under the curve; CI = confidence interval. ^a^ Histology counts provided for n = 266 enrolled subjects in Teschendorff, 2009 but analysis was limited to n = 235 cases. ^b^ AUC was estimated using the top 100 significant CpG probes for Teschendorff et al. and the top 6 CpG probes in Li et al. ^c^ AUC for pre-treatment cases with active disease (CA-125 serum > 30). ^d^ AUC for post-treatment cases with active disease (CA-125 serum > 30). ^e^ Subjects are a subset from Fridley, 2014 and are not counted in total sample size.

**Table 3:** DNA methylation associated with genome-wide significant genetic risk loci.

Study	Locus	Genetic Risk				DNA Methylation				
		SNP ^a^ (Gene)	Histotype	OR (95% CI)	*p*-Value	CpG Site ^b^	Correlated Expression	Histotype ^c^	Case Status	*p*-Value
Shen, 2013	17q12	rs7405776 (HNF1B)	Serous	1.13 (1.09–1.17)	3.1 × 10^−10^	cg14487292	*HNF1B*	Serous	Hyper	NE
		rs11651755 (HNF1B)	Clear cell	0.77 (0.70–0.84)	1.6 × 10^−8^			Clear cell	Hypo	NE
Yang. 2019	2q31.1	rs2072590 (HAGLR)	Serous	1.20 (1.14–1.25)	3.8 × 10^−14^	cg25137403	*HOXD4*	Serous	Hyper	9.1 × 10^−14^
		rs711830 (HOXD3)	Mucinous	1.30 (1.20–1.40)	7.5 × 10^−12^			Mucinous	Hyper	9.7 × 10^−9^
		rs2072590 (HAGLR)	Endometrioid	1.13 (1.04–1.22)	2.4 × 10^−3^			Endometrioid	Hyper	5.8 × 10^−5^
	3q25	rs7651446 (TIPARP)	All	1.44 (1.35–1.53)	1.5 × 10^−28^	cg26405475	*SSR3*	HG Serous	Hypo	1.9 × 10^−26^
	7p22.3	NA				cg03634833	*ADAP1*	All	Hypo	5.8 × 10^−7^
	8q24.21	rs10088218 (LINC00824)	Serous	0.76 (0.70–0.81)	8.0 × 10^−15^	cg08478672	*NA*	All	Hyper	3.8 × 10^−7^
	9q34.2	rs635634	All	1.11 (1.07–1.16)	4.4 × 10^−9^	cg14653977	*GBGT1 ABO*	All	Hyper	2.0 × 10^−9^
	10p12	rs1243180 (MLLT10)	All	1.10 (1.06–1.13)	1.8 × 10^−8^	cg04231319	*MLLT10*	All	Hypo	1.1 × 10^−8^
	17q21.31	rs2960000 (PLEKHM1)	Serous	1.16 (1.12–1.20)	3.3 × 10^−10^	cg07067577	*ARHGAP27MAPT*	All	Hypo	6.9 × 10^−14^
								HG Serous	Hypo	1.4 × 10^−10^
	17q21.32	rs9303542 (SKAP1)	All	1.12 (1.08–1.16)	6.0 × 10^−11^	cg19139618	*SKAP1 HOXB3 HOXB8*	All	Hypo	7.1 × 10^−7^
	19p13.11	rs2363956 (ANKLE1)	Serous	1.16 (1.11–1.21)	3.8 × 10^−11^	cg21956434	*ABHD8*	HG Serous	Hyper	5.7 × 10^−20^

All = All invasive EOC; Serous = High and low grade serous histotypes; Mucinous = Borderline/LMP and invasive mucinous histotypes; HG = High-grade. ^a^ First genome-wide significant SNP results reported and referenced. Gene reported is closest gene. ^b^ Top CpG site reported when more than one significant CpG was associated with a locus. ^c^ Histotype-specific results included for loci with significant differential associations for histotypes (by Cochran test).

## Data Availability

No new data were created or analyzed in this study. Data sharing is not applicable to this article.

## Supplementary Materials

The following are available online at https://www.mdpi.com/2072-6694/13/1/108/s1, Table S1: All DMPs identified through EWAS in EOC, Table S2: Genes replicated across EWAS in EOC.
